# Breast metastasis of signet ring cell carcinoma from the colon: a case report

**DOI:** 10.1186/s12957-022-02840-7

**Published:** 2022-12-01

**Authors:** Xiao Wang, Haibo Zhang, Yanwei Lu

**Affiliations:** 1 Cancer Center, Department of Medical Oncology, Zhejiang Provincial People’s Hospital, Affiliated People’s Hospital, Hangzhou Medical College, Hangzhou, Zhejiang China; 2 Cancer Center, Department of Radiation Oncology, Zhejiang Provincial People’s Hospital, Affiliated People’s Hospital, Hangzhou Medical College, Hangzhou, Zhejiang China

**Keywords:** Colon cancer, Breast metastasis from colon, Mucinous adenocarcinoma, Signet ring cell carcinoma, Case report

## Abstract

**Background:**

Colon cancer is one of the most common diagnosed malignancies. Despite the use of surgery, chemotherapy, radiotherapy, targeted therapy, immunotherapy, and other comprehensive treatments, distant metastasis is still one of the main causes for dying of colon cancer. The common metastatic site of colon cancer is the liver, lung, and bone. In this article, we report a rare case of breast metastasis of signet ring cell carcinoma from the colon.

**Case presentation:**

A 44-year-old woman was diagnosed with colon cancer and received a radical surgery of colon cancer in 2019. Combined with postoperative pathological and computed tomography (CT) images, a diagnosis of cT3N2M0 mucinous adenocarcinoma of colon (according to AJCC cancer staging manual, Version 8) was established. Adjuvant chemotherapy (XELOX: oxaliplatin 130 mg/m^2^ on day 1 plus capecitabine 1000 mg/m^2^ twice daily on days 1 to 14 every 3 weeks for 18 weeks) was performed followed by surgical resection. Fourteen months later, the patient underwent mastectomy for breast mass, which was diagnosed pathologically as metastasis of signet ring cell carcinoma from the colon. XELOX chemotherapy regimen (oxaliplatin 130 mg/m^2^ on day 1 plus capecitabine 1000 mg/m^2^ twice daily on days 1 to 14 every 3 weeks for 24 weeks) combined with bevacizumab (7.5 mg/kg on day 1) was used after the mastectomy. The patient had stable disease according to her last examination (RECIST criteria).

**Conclusion:**

It is rare to find a report of a patient of colon cancer that metastasizes to breast. We hope to increase treatment experience for patients with this rare metastasis.

## Background

Colon cancer is the third most common diagnosis malignancy worldwide [1, 2]. Nowadays, the main treatment methods of colon cancer include surgical resection, chemotherapy, radiotherapy, targeted therapy, and immunotherapy [3]. With the help of those treatments, 5-year survival rates for patients with localized or regionally disease is 90.1% and 69.2%, respectively. But the 5-year survival rate is only 11.7% for patients suffering from colon cancer metastasis [2]. Colon cancer often metastasizes to the liver, lung, or bone; metastasis to the breast is not common [4]. In this article, we report a rare case of breast metastasis from colon cancer.

## Case presentation

**Fig. 1:**
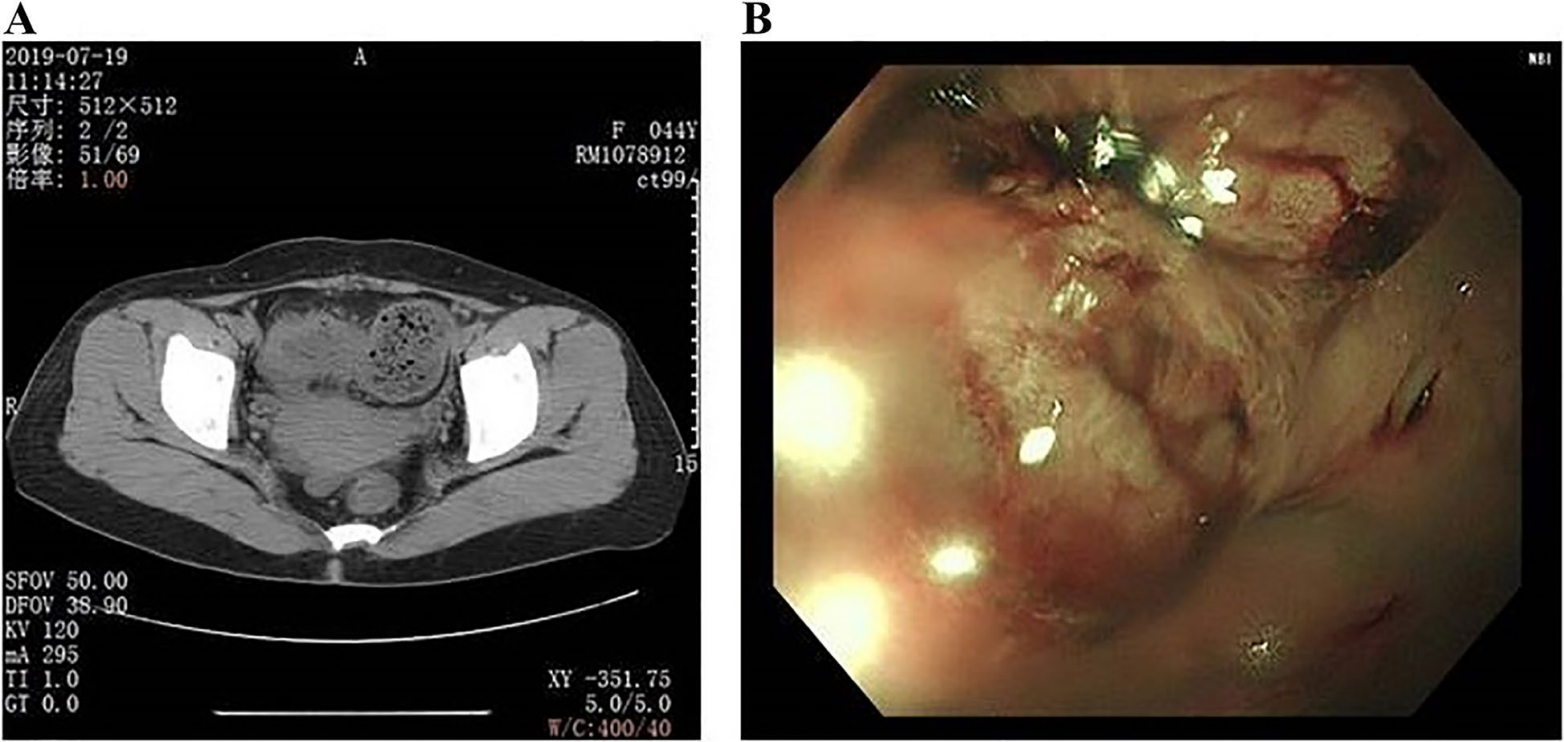
**A** Abdominal computed tomography (CT) showed thickening of intestinal wall at the junction of sigmoid colon and descending colon. **B** Colonoscopy revealed a colonic mass

**Fig. 2:**
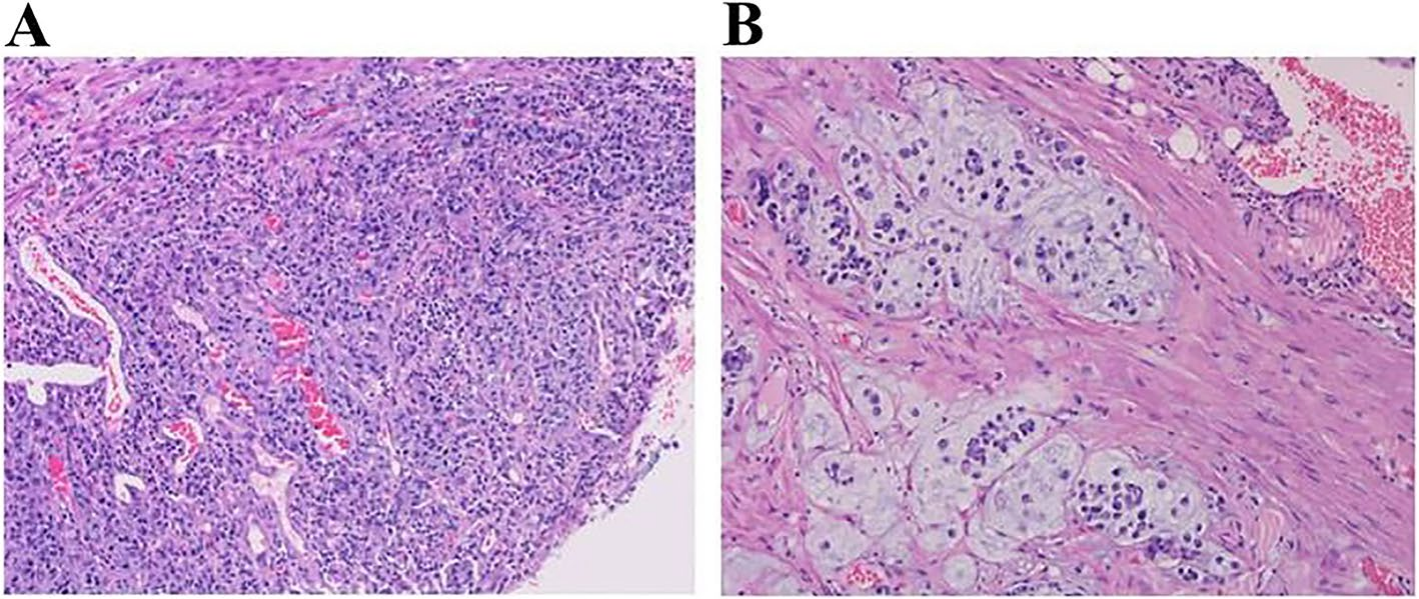
Microscopy examination of the colon specimen. **A** Original magnification × 20. **B** Original magnification × 200

A 44-year-old woman was initially referred to local hospital for recurrent left abdominal obtuse pain without obvious cause. She had no surgical history or chronic diseases, such as hypertension or diabetes. She denied family genetic history and tumor history. Her performance status was normal with 37.0 °C of initial body temperature, 89 beats/min of pulse rate, 20 breaths/min of breath rate, and 130/79 mmHg of blood pressure. A physical examination revealed left abdominal tenderness, but there was no muscle guarding or rebound tenderness. No obvious mass was palpable in the abdomen, and bowel sounds were normal. An abdominal CT showed feces in the colon, thickening of the intestinal wall at the junction of sigmoid colon, and descending colon (Fig. 1A). Colonoscopy revealed colonic stenosis is 60-cm away from the anus, which may on account of colonic mass or colitis (Fig. 1B). Blood tests showed that the patient’s CA72.4 was 11.48 U/mL (normal reference value: 0-6.90). Laparoscopic radical resection was performed. Pathological examination showed as sigmoid colon cancer, which was poorly differentiated mucinous adenocarcinoma with serosa invasion, vessel invasion, and 4 of 10 peri-intestinal lymph node metastasis (Fig. 2). Gene test results for KRAS, NRAS, BRAF, and exon15 V600E were negative. According to the AJCC cancer staging manual (Version 8), the patient was considered as stage of IIIB with cT3N2M0. Adjuvant chemotherapy regimen of XELOX (oxaliplatin 130 mg/m^2^ on day 1 plus capecitabine 1000 mg/m^2^ twice daily on days 1 to 14 every 3 weeks for 18 weeks) was used followed by surgical resection. After the 6 cycles of adjuvant chemotherapy finished, the patient was followed up every 3 months. And the examination results showed that the patient’s disease was stable.

**Fig. 3:**
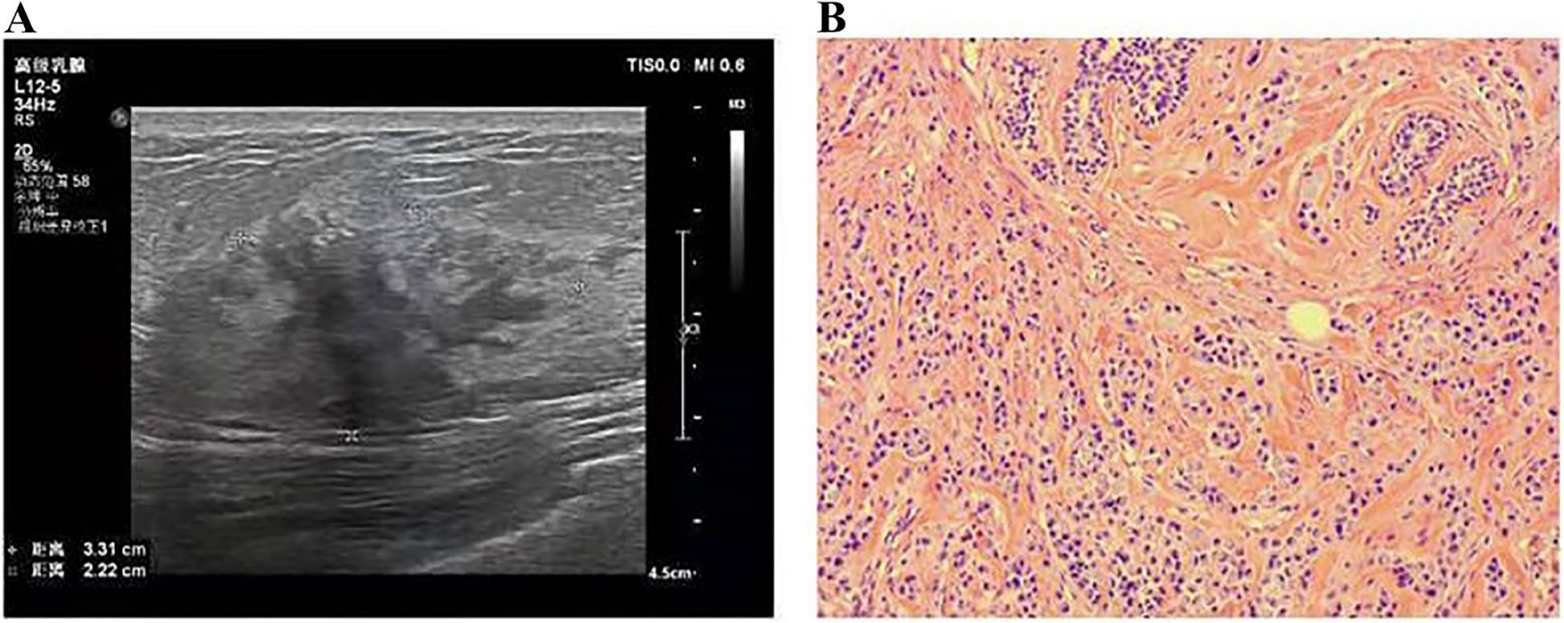
**A** Ultrasound detected an irregular-shaped hypoechoic right breast mass of 33 × 23 mm size. **B** Microscopy examination of the breast specimen, original magnification × 200

**Fig. 4:**
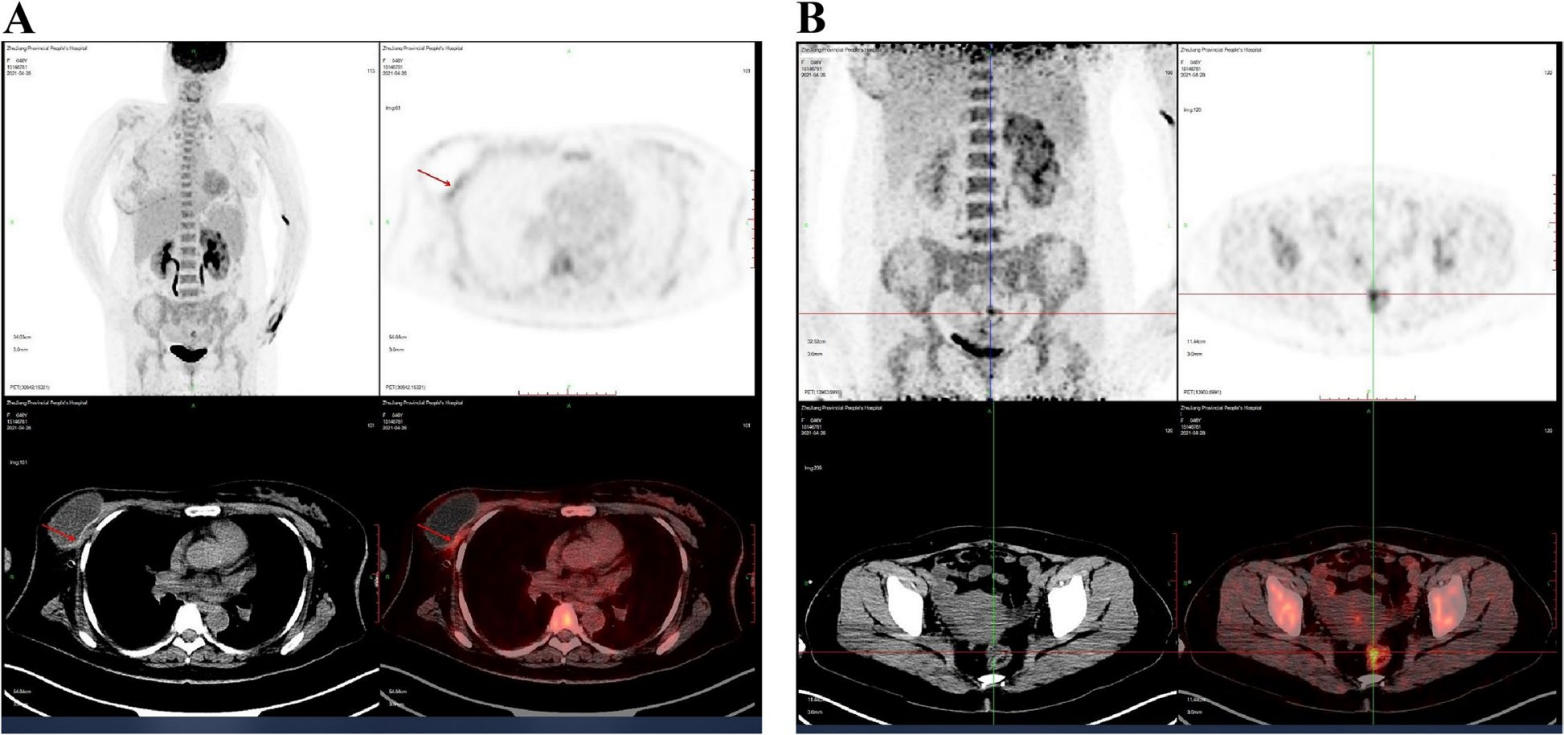
Positron emission tomography computed tomography (PET/CT) after mastectomy. **A** A strip soft tissue density signal was detected in the right breast surgical area, where a prosthesis was implanted. The maximum standardized uptake (SUVmax) of 18F-FDG was 5.3. **B** After the radical resection of colon cancer, ^18^F-FDG was increased at the anastomotic site and distal intestine with a SUVmax value of 4.4. Tracer distribution was enhanced at transverse colon, splenic flexure of colon, and rectum. The SUVmax of ^18^F-FDG was 6.6. Hysteromyoma could also be seen on the image

The patient presented with breast tenderness after 14 months diseases-free interval. Physical examination revealed a mass of 3.2 × 2.0 cm size on the inner upper quadrant of the right breast. Ultrasonography showed an irregular mass of 33 × 23 mm size in the right breast with unclear boundary (Fig. 3A). Breast biopsy displayed that the mass was an invasive lobular carcinoma. The patient underwent modified radical mastectomy for her disease. The postoperative immunohistochemistry of the mass showed as the following: ER, PR, HER-2 results negative; CK-pan, CK-20, CEA results positive; P120, CDX-2 results weakly positive; Ki67 approximately expressed 8%; E-cad, CK-7 results negative. Pathological examination demonstrated that the mass was a metastatic signet ring cell carcinoma, which came from the intestinal tract. Tumor tissue invaded the para-mammary adipose tissue. And the cancer tissue was discovered in the vessel. For further treatment, the patient went to our hospital. Considering the rarity of breast metastasis from the colon cancer, we invited the pathologist in our hospital to consult the pathological sections of this patient. Suggestion of pathological consultation showed that GATA3, mammaglobin, GCDFP-15, HER2, ER, PR, and CDK5/6 were negative; E-cad expressed weakly positive; CDX-2 resulted positive; and Ki67 expressed 10%, which is considered as metastatic signet ring cell carcinoma (Fig. 3B). Positron emission tomography computed tomography (PET/CT) exhibited strip soft tissue density signal in the surgical area with a prosthesis implantation. The maximum standardized uptake (SUVmax) of ^18^F-FDG was 5.3, which was considered as the postoperative changes of breast (Fig. 4A). After the radical resection of colon cancer, ^18^F-FDG was increased at the anastomotic site and distal intestine with a SUVmax value of 4.4. Tracer distribution was enhanced at the transverse colon, splenic flexure of colon, and rectum. SUVmax of ^18^F-FDG was 6.6, which was considered as physiological metabolism and may complicate with intestinal polyps (Fig. 4B). Hysteromyoma also could be seen on the image (Fig. 4B). Adjuvant chemotherapy based on 130 mg/m^2^ oxaliplatin on day 1 plus 1000 mg/m^2^ capecitabine twice daily on days 1 to 14 and combined with 7.5 mg/kg bevacizumab on day 1 every 21 days for 8 cycles was used for this patient. The last re-examination showed that the patient had stable disease (RECIST criteria).

## Discussion

The major histological type of colon cancer is adenocarcinoma, which accounts for more than 90% of all cases [3]. Mucinous adenocarcinomas represent about 8–10% of colon cancer [5]. Signet-ring cell carcinoma is a rare separate classification, which accounts for 2–4% of mucinous carcinomas and characters by containing intracellular mucin pushing the nucleus to one side [3]. Both mucinous adenocarcinomas and signet-ring cell carcinoma represent aggressive behavior and were related to poor prognosis in patients [6, 7]. Recent research demonstrated that female and younger patients are more likely to suffer from mucinous adenocarcinoma, which is more frequently diagnosed at an advanced stage [8]. In this article, we report a case of 44-year-old Chinese woman who suffered from stage of IIIB with mucinous adenocarcinoma of colon and metastatic signet ring cell carcinoma of breast, which is consistent with the characteristics of middle-aged women prone to this disease. Covering more than 50% of the mucinous component, mucinous adenocarcinoma is composed of an extracellular mucin pool, which may contain layers, acini, cribriform sheets of malignant glands, or scattered individual signet ring cells [9]. Signet ring cell carcinoma was classified as mucinous adenocarcinoma with more than 50% signet ring cell component [10]. Due to the different proportion of signet ring cells, colon cancer contains both mucinous and signet ring cell components are occasionally confusing. This may explain why the pathological results of colon and breast in this case are inconsistent. Moreover, immunohistochemistry for cytokeratin 20 (CK-20) and caudal-type homeobox 2 (CDX2) can accurately identify colon adenocarcinoma origin [3]. Cytokeratin 7 (CK-7) is positive for most breast cancers but negative for colon cancer [11]. In addition, GATA3, mammaglobin, GCDFP-15, and ER are most commonly used markers to identify breast origin [12]. In this case, the patient’s immunohistochemistry of the breast showed that CK-20 positive expression, CDX-2 weakly positive expression and CK-7, GATA3, mammaglobin, GCDFP-15, and ER negative expression, which confirmed the intestinal tract origin. Due to the lack of typical clinical symptoms, signs, and imaging findings, it is difficult to distinguish primary or metastatic breast cancer. We believe that specific immunohistochemical markers are helpful for more accurate diagnosis.

Breast tumors are mostly primary cancers in women, but tumors metastasis to breast is quite rare, which account for only 0.5–3% of all breast metastasis [13]. Although literature reported some rare causes of metastasis to breast, such as contralateral breast, ovary, lung, stomach, leukemia, melanoma, and lymphoma; colon metastasis to breast was even more rare [14–16]. As far as we know, there are no more than 30 cases of colon cancer metastasis to breast worldwide [13, 17, 18]. Researchers’ statistical analysis of those cases revealed that the average time form colorectal cancer diagnosis to breast metastasis was 21 months, and the longest transfer time to breast was 7 years so far [13]. After detection, the average survival time is 14.9 months [17]. Only one case had a more than 5-year overall survival with colon cancer metastasis to breast [17]. As shown in our case, histopathology often exhibited mucinous or signet-ring cell features for those patients [13]. It is well known that dissemination of clonogenic cells lead to the formation of the micrometastatic foci; some of those clonogenic cells had characteristics that are similar with the primary tumor. By systemic circulation, lymphatic circulation, or transcoelomic migration, these clonogenic cells spread [14]. However, this theory could not explain the solitary metastasis of rare sites, such as breast. Baum and his colleagues proposed a hypothesis that primary cancer cells shed subcellular particles, which were taken up by wandering cells of the monocyte macrophage system and transported to distant sites. Subsequently, the genetic information in subcellular particles was transfected to the local mesenchymal cells. Thus, the expression of oncogenic sequences and the development of cancer cell phenotypes occur in usual locations [19].

Surgery is the main treatment for colon cancer, but tumors recur in 30–50% of all cases, which usually present as metastasis [20]. After surgery resection, adjuvant chemotherapy is the standard treatment for patients with stage III colon cancer, which could provide a 22 to 32% overall survival advantage and a 30% relative risk reduction in recurrence [21]. For patients with unresectable locally advanced disease or high metastatic burden, palliative systemic chemotherapy is appropriate. And patients with individualized local-recurrent disease may receive multimodality therapy. In general, there is a lack of experience in treatment of patients with rare-site metastasis. Goel and his colleagues reported an unusual case of locally advanced rectal cancer metastasizes to eyelid, which received preoperative radiotherapy and diversion colostomy for primary tumor [22]. When eyelid metastasis occurred, the patient treated with three cycles of weekly chemotherapy and was planned for excision and reconstruction of eyelid [22]. Unfortunately, the patient eventually died of small bowel obstruction, acute renal failure, and septicemia with an overall duration of survival of 7 months [22]. For now, a majority of the patients with breast metastasis form colon received standard treatment as their primary tumor [23]. If the patients recur with solitary nodules within the breast, surgical excision with negative margins may benefit for them [24, 25]. To slow the growth of breast metastasis, patients accepted oral capecitabine as palliative chemotherapy [13, 17]. Simple mastectomy was needed for bulky or painful tumor [15]. Avoiding surgical excision and giving systemic chemotherapy is an option for patients with short survival and poor prognosis. [26, 27]. Combination capecitabine and bevacizumab may be helpful to elderly patients with breast metastasis from colon [17]. In the patient we are describing, she underwent radical surgery for stage IIIB of primary colon cancer followed by 6 cycles of XELOX adjuvant chemotherapy regimen. Unfortunately, she presented breast metastasis 14 months later. Because of the solitary nodules within the right breast, the patient underwent mastectomy with negative margins. Considering her younger age, more aggressive pathology, and primary tumor, we performed XELOX chemotherapy regimen combination with bevacizumab to this patient for 8 cycles. Clinical examination after the last multimodality therapy showed as stable disease (RECIST criteria).

Breast metastasis form primary colon cancer is rare. The mechanism of this metastasis has not been fully clarified, and the prognosis of this disease is poor. At present, there is no unified standard treatment for this disease. Through the sharing of this case, we hope to increase the knowledge for breast metastasis form primary colon cancer and provide an effective treatment mode for this disease.

## Conclusion

Our report represented a rare case of breast metastasis form primary colon cancer. We provided an effective treatment regimen which combined surgery, chemotherapy, and target therapy. We will continue to follow up the prognosis of patients.

## Data Availability

All information about the patient came from oncology center, Zhejiang Provincial People’s Hospital, People’s Hospital of Hangzhou Medical College. All data generated or analyzed during this study are included in this published article.

## Abbreviations

CT: Computed tomography
XELOX: Oxaliplatin 130 mg/m^2^ on day 1 plus capecitabine 1000 mg/m^2^ twice daily on days 1 to 14 every 3 weeks for 18 weeks
CA 724: Carbohydrate antigen 724
AJCC: American Joint Committee on Cancer
KRAS: Kirsten rat sarcoma viral oncogene homolog
NRAS: Neuroblastoma RAS viral (v-ras) oncogene homolog
BRAF: V-raf murine sarcoma viral oncogene homolog B1
CK-pan: Cytokeratin-pan
CK-20: Cytokeratin-20
CK-7: Cytokeratin-7
CEA: Carcinoembryonic antigen
Ki-67: Marker of proliferation Ki-67
P120: P120 catenin
CDX-2: Caudal-type homeobox protein 2
E-cad: E-cadherin
GATA3: GATA binding protein 3
GCDFP-15: Gross cystic disease fluid protein 15
HER2: Human epidermal growth factor receptor 2
ER: Estrogen receptor
PR: Progesterone receptor
CDK5/6: Cyclin-dependent kinase 5/6
PET/CT: Positron emission tomography computed tomography
SUVmax: Maximum standardized uptake
^18^F-FDG: ^18^Fluorine-fluorodeoxyglucose
RECIST: Response Evaluation Criteria in Solid Tumors
